# Surgical indications for pleurectomy/decortication in pleural mesothelioma based on the newly revised 9th edition of the tumour–node–metastasis classification

**DOI:** 10.1093/icvts/ivae223

**Published:** 2024-12-28

**Authors:** Masatoshi Kanayama, Masaru Takenaka, Takehiko Manabe, Katsuma Yoshimatsu, Rintaro Oyama, Hiroki Matsumiya, Masataka Mori, Koji Kuroda, Fumihiro Tanaka

**Affiliations:** Second Department of Surgery, University of Occupational and Environmental Health, Japan, Yahatanishi-ku, Kitakyushu, Japan; Second Department of Surgery, University of Occupational and Environmental Health, Japan, Yahatanishi-ku, Kitakyushu, Japan; Second Department of Surgery, University of Occupational and Environmental Health, Japan, Yahatanishi-ku, Kitakyushu, Japan; Second Department of Surgery, University of Occupational and Environmental Health, Japan, Yahatanishi-ku, Kitakyushu, Japan; Second Department of Surgery, University of Occupational and Environmental Health, Japan, Yahatanishi-ku, Kitakyushu, Japan; Second Department of Surgery, University of Occupational and Environmental Health, Japan, Yahatanishi-ku, Kitakyushu, Japan; Second Department of Surgery, University of Occupational and Environmental Health, Japan, Yahatanishi-ku, Kitakyushu, Japan; Second Department of Surgery, University of Occupational and Environmental Health, Japan, Yahatanishi-ku, Kitakyushu, Japan; Second Department of Surgery, University of Occupational and Environmental Health, Japan, Yahatanishi-ku, Kitakyushu, Japan

**Keywords:** Surgical indications, Pleural mesothelioma, The 9th edition of the TNM classification

## Abstract

**OBJECTIVES:**

The MARS2 trial questioned the efficacy of curative intent surgery for pleural mesothelioma (PM), while real-world clinical data from Japan suggest a favourable prognosis in surgical cases, indicating survival benefits in selected patients. The newly revised 9th edition of the tumour–node–metastasis (TNM) classification introduces a novel indicator based on pleural thickness.

**METHODS:**

We conducted a retrospective evaluation of patients with PM who underwent pleurectomy/decortication between 2012 and 2022. Patient characteristics, complications and treatment outcomes were assessed. Additionally, outcomes were analysed based on the 9th edition of the TNM classification.

**RESULTS:**

A total of 62 patients were included in the analysis. The median overall survival (OS) was 37.3 months, with a median relapse-free survival (RFS) of 15.5 months. Patients with the epithelioid subtype (OS: 61.6 months; RFS: 26.0 months) and pStage IA (OS: not reached; RFS: 69.1 months) had significantly better outcomes. The 9th edition of the TNM classification showed a stronger prognostic correlation than the 8th edition, with a median OS of 77.0, 31.9, 20.4 and 25.3 months for stages I, II, IIIA and IIIB (*P* = 0.0016) and median RFS of 34.3, 12.3, 13.7 and 6.9 months for stages I, II, IIIA and IIIB (*P* = 0.013), respectively.

**CONCLUSIONS:**

Surgical intervention remains crucial in the treatment of PM, particularly for patients with epithelioid histology and early stages of the disease. This study evaluates surgical indications for PM using the newly revised 9th edition of the TNM classification, indicating that it enhances the precision of surgical candidate selection and potentially improves patient outcomes.

## GRAPHICAL ABSTRACT

**Figure:**
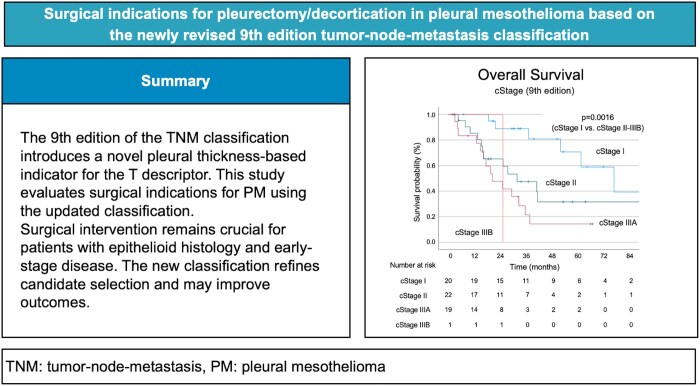

## INTRODUCTION

Pleural mesothelioma (PM) is a highly aggressive disease with a poor prognosis. Current guidelines emphasize the importance of multimodal treatment for PM, suggesting curative intent surgery for selected patients with resectable early-stage disease [1]. However, at the 2023 World Lung Cancer Congress, results from the MARS2 trial were negative for curative intent surgery in PM, showing a median survival of 19.3 months in the surgery group compared to 22.4 months in the non-surgery group [2]. Even the epithelioid subtype, which was previously considered to benefit from surgery, did not demonstrate any additional survival advantage from surgical intervention. This finding has a significant impact on PM treatment strategy. However, interpretation of these results is complex due to several factors, including the considerable proportion of patients presenting with advanced T stage (T3: 34.3%) and nodal involvement (N-positive: 27.8%), low rates of macroscopic complete resection (MCR) and relatively elevated perioperative mortality rates (30-day mortality: 3.8%, 90-day mortality: 8.9%).

In contrast, real-world data from Japan demonstrate a median overall survival (OS) of 32.2 months among surgically treated patients with PM, a significant improvement compared to the 14.0 months observed in non-surgically treated patients [3]. The study reveals that 87.7% of cases were diagnosed at cStage I (IA: 55.8%; IB: 31.9%), indicating a predominance of early-stage disease. Moreover, the perioperative mortality rates were notably low, with 30- and 90-day mortality rates of 0.7% and 4.3%, respectively. These findings highlight the importance of appropriate patient selection, meticulous perioperative management and advanced surgical techniques in achieving significant survival benefits.

In the newly revised 9th edition of the tumour–node–metastasis (TNM) classification, a novel indicator based on pleural thickness for the T descriptor [4] has been introduced. Thus, a re-evaluation of the selection criteria for surgery is required to improve outcomes. This study systematically compiled and analysed the treatment outcomes of curative intent surgery [pleurectomy/decortication (P/D)] performed at our institution, elucidating the implications of these findings in surgical indications.

## PATIENTS AND METHODS

### Participants and study design

This retrospective study was approved by the Ethics Committee of the University of Occupational and Environmental Health, Japan (approval number: 19-042). Informed consent was not required for this observational study. Instead, patients were informed about the study, and they had the option to opt out if they did not wish to participate. This approach was approved by the Ethics Committee. Between January 2012 and December 2022, P/D procedures were performed on 67 patients at our institution. Cases involving salvage surgery following immune checkpoint inhibitor treatment (*n* = 5) were excluded, leaving 62 cases for retrospective analysis.

Preoperative evaluation included assessment of the Eastern Cooperative Oncology Group performance status, blood tests, cardiac and respiratory function assessments and imaging examinations (radiographs, contrast-enhanced computed tomography and positron emission tomography-computed tomography). According to the 8th edition of the International Mesothelioma Interest Group classification, surgery was typically indicated for clinical stage T1-3N0-1M0, with a performance status of 0–1, and was generally applicable for patients with the epithelioid subtype [5]. Our treatment strategy for resectable PM involves P/D [6, 7] followed by postoperative adjuvant therapy. Prior to surgery, pleurodesis using OK-432 was typically performed to promote pleural adhesion and prevent postoperative complications. Extended P/D was performed based on intraoperative findings when MCR could not be achieved without resection of adjacent structures due to the extent of tumour invasion. Perioperative complications were graded according to the Clavien–Dindo classification [8], with events of grade ≥2 considered clinically meaningful for inclusion in our analysis. Prolonged air leakage was defined as the need to extend chest tube drainage for more than 7 days or to perform interventions such as pleurodesis. Pathological staging was determined based on intraoperative findings and postoperative examination of surgical specimens.

### New T-descriptor evaluation (Psum, Fmax) introduced in the 9th edition of the TNM classification

Based on the newly introduced 9th edition [4], the measurement of pleural thickness was conducted by 2 independent thoracic surgeons who were unaware of any clinical information. In instances of significant discrepancies between the 2 assessments, simultaneous examination was conducted to reach a consensus. Psum refers to the sum of 3 measurements of maximal pleural thickness on axial images, taken along the chest wall or mediastinum in each of the 3 chest divisions—upper, middle and lower—delineated by 2 lines: one at the top of the aortic arch and the second at the top of the left atrium. Fmax represents the maximal thickness of pleural tumour along the fissures, measured on sagittal images.

### Statistical analysis

Patient characteristics were summarized using medians and ranges for continuous variables and frequencies for categorical variables. OS was calculated from the date of surgery until death or the last follow-up, while relapse-free survival (RFS) was calculated from the date of surgery until relapse, death or last follow-up. The Kaplan–Meier method was employed to estimate OS and RFS, with 95% confidence interval (CI) provided for the survival estimates. Survival differences between groups were compared using the log-rank test. A multivariable analysis of prognostic factors was performed using a Cox proportional hazards regression model. Statistical significance was set at *P* < 0.05. Receiver operating characteristic curve analysis was conducted to evaluate the prognostic accuracy of the multivariable Cox regression model. The area under the curve (AUC) was calculated for both OS and RFS. AUC values were reported with 95% CI to assess the robustness of the model. All analyses were performed using SPSS (version 28.0, IBM Corp., Armonk, NY, USA).

## RESULTS

### Patient characteristics

The median age was 69 years (range, 47–82 years), with a male predominance (56 males and 6 females). Most tumours were located on the right side of the thorax (71.0%). Histological subtypes comprised 46 cases of epithelioid, 6 biphasic and 10 sarcomatoid PM. Clinical staging (8th edition) was IA in 30, IB in 24, II in 6 and IIIB in 2 cases (Table 1). All patients underwent P/D, while extended P/D, including pericardial or diaphragmatic resection, was performed in 55 cases (88.7%). The median operative time was 382 min (range, 204–884 min), and the median blood loss was 1695 ml (range, 420–9100 ml). The rate of MCR was 91.9% (57/62). Postoperative complications occurred in 75.8% (47/62) of cases, with a complication rate of 43.5% (27/62) after excluding prolonged air leaks. There were no deaths 30 days postoperatively, while one death was recorded within 90 days postoperatively. In this study, neoadjuvant chemotherapy was administered to 7 patients: 3 were referred to our institution after starting treatment at other hospitals, 2 received neoadjuvant therapy due to preoperative N1 disease, 1 due to suspicion of T4 disease and 1 due to scheduling conflicts for surgery. Pathological staging (8th edition) distributed as IA in 9, IB in 32, II in 2, IIIA in 8 and IIIB in 11 cases. Postoperative adjuvant chemotherapy was administered to 46 patients (76.7%, unknown: 2 cases) (Table 2).

**Table 1:** Patients’ characteristics

Characteristics	Patients (*n* = 62)
Age (years), median (range)	69 (47–82)
Sex	
Male	56 (90.3%)
Female	6 (9.7%)
ECOG PS	
0–1	61 (98.4%)
2	1 (1.6%)
Tumour location	
Right	44 (71.0%)
Left	18 (29.0%)
Histological subtype	
Epithelioid	46 (74.2%)
Sarcomatoid	10 (16.1%)
Biphasic	6 (9.7%)
Clinical T category (8th edition)	
T1	34 (54.8%)
T2	16 (25.8%)
T3	11 (17.7%)
T4	1 (1.6%)
Clinical stage (8th edition)	
IA/IB	30 (48.4%)/24 (38.7%)
II	6 (9.7%)
IIIA/IIIB	0 (0.0%)/2 (1.6%)
IV	0 (0.0%)
Pathological stage (8th edition)	
IA/IB	9 (14.5%)/32 (51.6%)
II	2 (3.2%)
IIIA/IIIB	8 (12.9%)/11 (17.7%)
IV	0 (0.0%)
Psum	
<12 mm	20 (32.3%)
12–30 mm	25 (40.3%)
>30 mm	17 (27.4%)
Fmax	
≤5 mm	59 (95.2%)
>5 mm	3 (4.8%)
Clinical T category (9th edition)	
T1	20 (32.3%)
T2	24 (38.7%)
T3	17 (27.4%)
T4	1 (1.6%)
Clinical stage (9th edition)	
I	20 (32.3%)
II	22 (35.4%)
IIIA/IIIB	19 (30.6%)/1 (1.6%)
IV	0 (0%)

ECOG PS: Eastern Cooperative Oncology Group performance status; Fmax: maximal thickness of pleural tumour along the fissures measured on sagittal images; Psum: sum of 3 measurements of maximal pleural thickness measured on axial images.

**Table 2:** Operation and postoperative course

	Patients (*n* = 62)
Combined resection	55 (88.7%)
Pericardium	39 (71.0%)
Diaphragm	55 (100.0%)
Lung	12 (21.8%)
Resectability status	
R1	57 (91.9%)
R2	5 (8.1%)
Bleeding volume (ml), median (range)	1695 (420–9100)
Operative time (min), median (range)	382 (204–884)
Postoperative complications	47 (75.8%)
Supraventricular tachycardia	13 (21.0%)
Pneumonia/atelectasis	8 (12.9%)
Prolonged/delayed pulmonary air leak	37 (59.7%)
Complications excluding pulmonary air leak	27 (43.5%)
Postoperative mortality	
Within 30 days	0 (0.0%)
Within 90 days	1 (1.6%)
Adjuvant chemotherapy	46 (76.7%)
Postoperative death	28 (45.2%)
Postoperative relapse	43 (69.4%)
Loco-regional	34 (79.1%)
Both loco-regional and distant	9 (20.9%)

Loco-regional relapse: relapse within the thoracic cavity, including the pleura, lung, pericardium, diaphragm, and along the surgical field (e.g. incision sites, chest wall).

Distant relapse: relapse occurring outside the thoracic cavity, such as in the abdominal cavity, peritoneum, liver, bones or distant lymph nodes.

### Survival analysis

**Figure 1:**
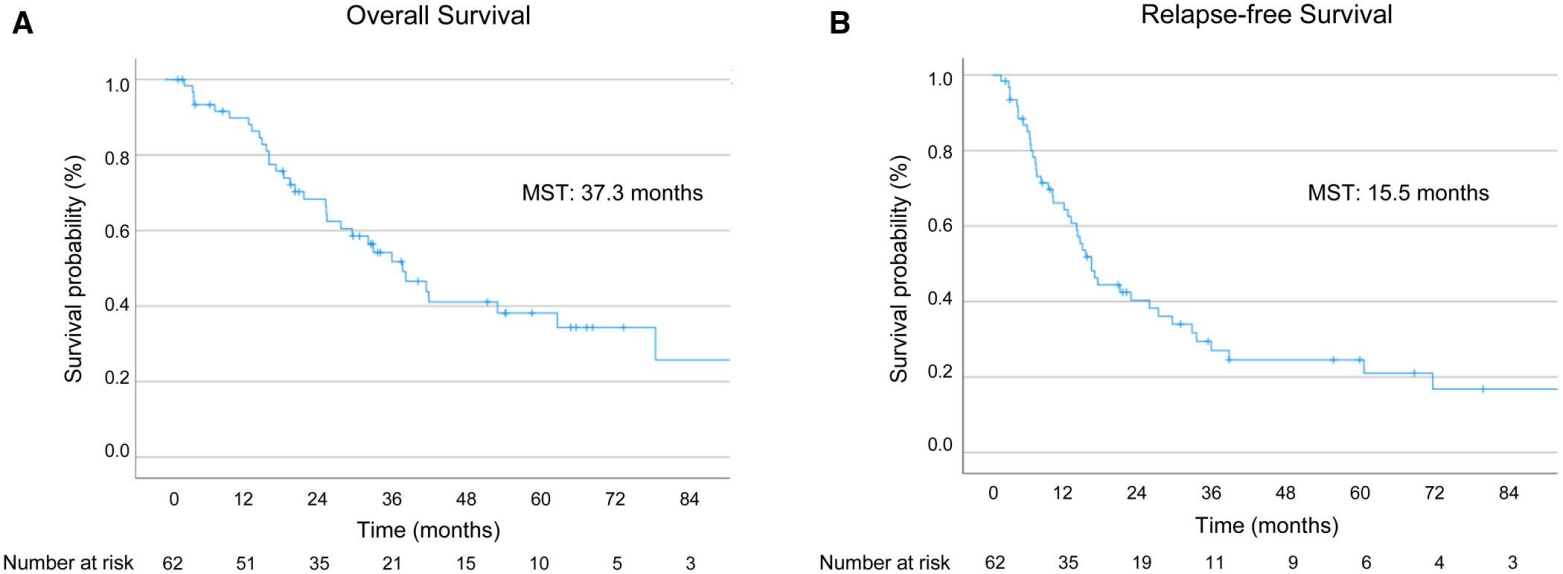
Overall survival (**A**) and relapse-free survival (**B**) after pleurectomy/decortication.

**Figure 2:**
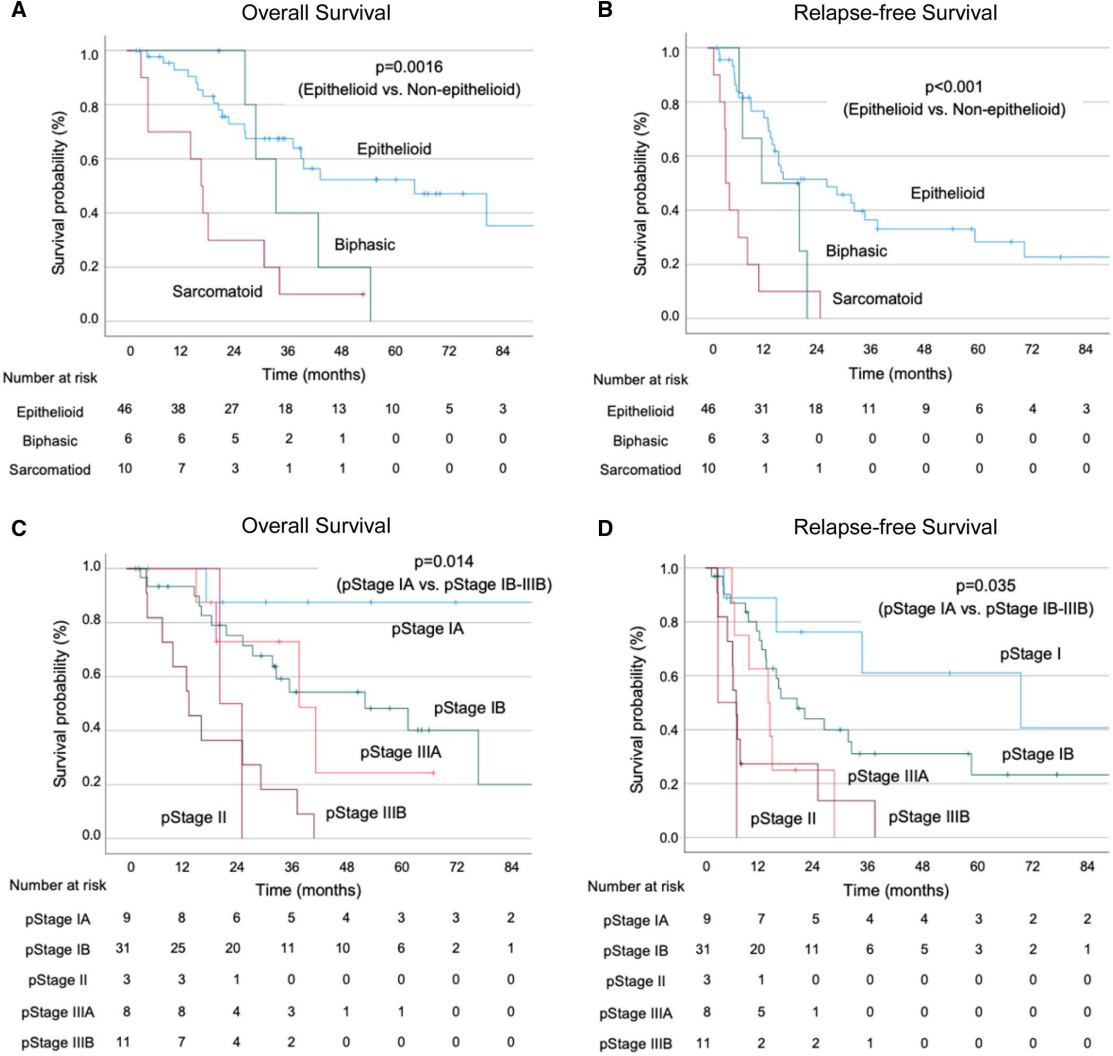
Overall and relapse-free survival after pleurectomy/decortication based on histological subtype (**A**, **B**) and pathological stage (8th edition) (**C**, **D**).

**Figure 3:**
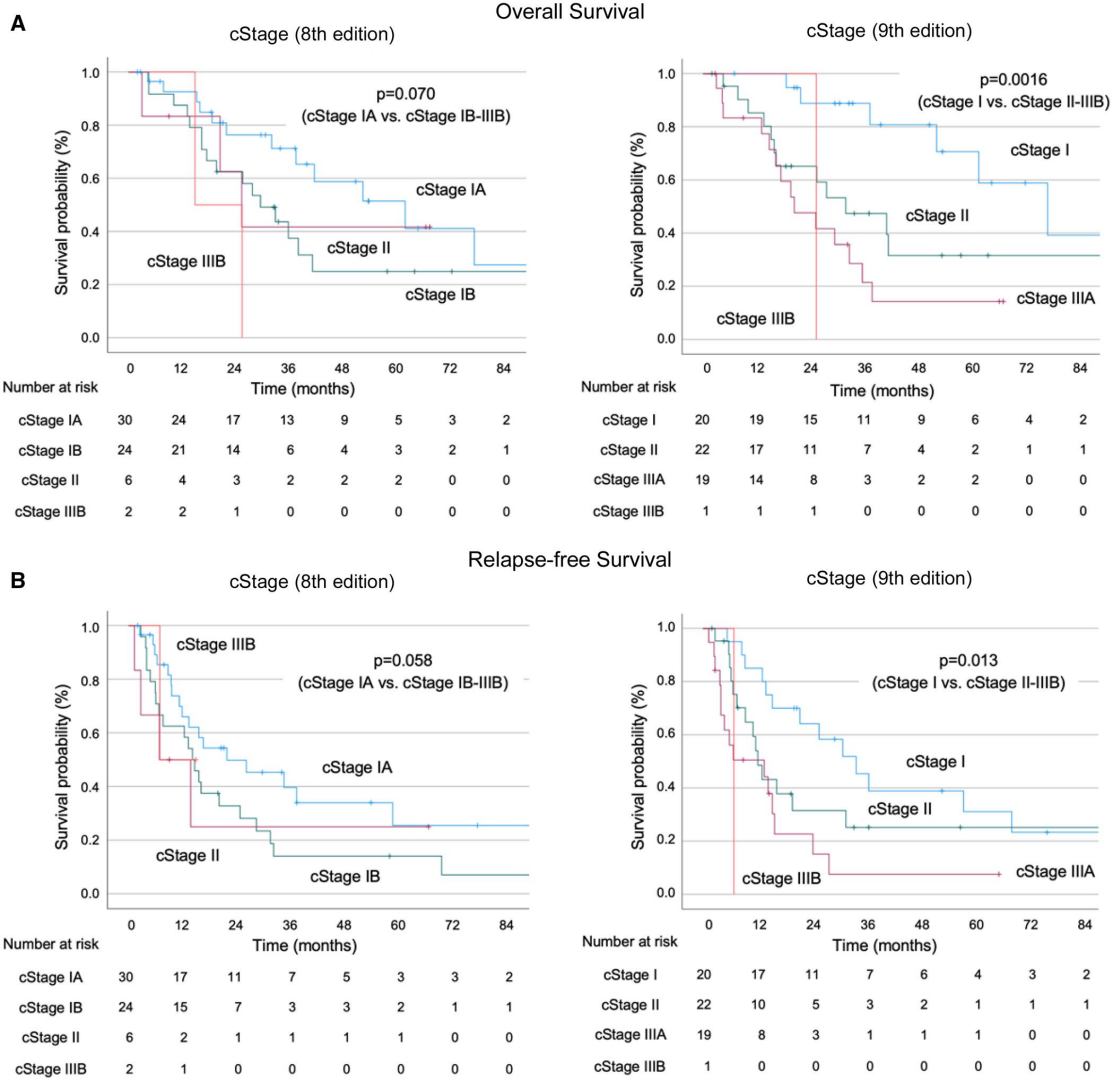
Overall survival (**A**) and relapse-free survival (**B**) after pleurectomy/decortication based on pathological stage (8th and 9th editions).

Among the 62 patients, 28 (45.2%) died during the follow-up period, and 43 (69.4%) experienced relapses. Of these, 34 (79.1%) had loco-regional relapse, and 9 (20.9%) experienced both loco-regional and distant relapse (3 cases in the liver, 2 cases with ascites, 2 cases in bone, 1 case in the abdominal lymph nodes and 1 case in the brain). The median follow-up period was 28.5 months (range, 2.0–152.9 months). The median OS for all patients was 37.3 months (95% CI 27.3–47.3 months), and the median RFS was 15.5 months (95% CI 12.2–18.8 months) (Fig. 1A and B). When analysed by histological subtype, the median OS was 61.6 months (95% CI 27.4–95.8 months), 31.9 months (95% CI 22.7–41.1 months) and 15.9 months (95% CI 11.7–20.1 months) for the epithelioid, biphasic and sarcomatoid subtypes, respectively. The median RFS was 26.0 months (95% CI 8.4–43.6 months), 11.8 months (95% CI 0–23.7 months) and 4.0 months (95% CI 3.0–5.0 months) for the epithelioid, biphasic and sarcomatoid PM, respectively, demonstrating significantly better outcomes for the epithelioid subtype compared to the non-epithelioid subtypes (OS: *P* = 0.0016, RFS: *P* < 0.001) (Fig. 2A and B). When analysis was performed by pathological stage (8th edition), the median OS was not reached for IA, 52.2 months (95% CI 20.7–83.7 months) for IB, 20.4 months (95% CI NA–NA) for II, 37.8 months (95% CI 17.9–57.7 months) for IIIA and 13.6 months (95% CI 6.9–20.2 months) for IIIB. The median RFS was 69.1 months (95% CI 0.3–137.9 months) for IA, 20.0 months (95% CI 10.8–29.2 months) for IB, 2.7 months for II (95% CI NA–NA), 13.7 months (95% CI 7.3–20.1 months) for IIIA and 6.7 months (95% CI 5.6–7.8 months) for IIIB. Our findings correlated with pathological stage, with favourable outcomes for early-stage cases, particularly pStage IA compared to pStage IB and beyond (OS: *P* = 0.014, RFS: *P* = 0.035) (Fig. 2C and D). When analysed by clinical stage (8th edition), the median OS was 61.6 months (95% CI 31.8–91.4 months) for IA, 29.4 months (95% CI 19.1–39.7 months) for IB, 25.2 months (95% CI 15.2–35.2 months) for II and 14.8 months (95% CI NA–NA) for IIIB. The median RFS was 21.7 months (95% CI 2.0–41.4 months) for IA, 14.1 months (95% CI 10.3–17.9 months) for IB, 6.8 months (95% CI 0–17.4 months) for II and 6.9 months (95% CI NA–NA) for IIIB. These findings indicate a trend towards better outcomes for cStage IA compared to cStage IB and beyond, although statistical significance was not reached (OS: *P* = 0.070, RFS: *P* = 0.058) (Fig. 3A and B).

### The impact of the 9th edition of the TNM classification

The clinical staging based on the newly introduced 9th edition is summarized in Table 1. According to this classification, there were 20 cases in stage I, 22 cases in stage II, 19 cases in stage IIIA and 1 case in stage IIIB. Using the 9th edition of the TNM classification, the median OS was 77.0 months (95% CI 47.8–106.2 months) for stage I, 31.9 months (95% CI 13.8–50.0 months) for stage II, 20.4 months (95% CI 10.0–30.8 months) for stage IIIA and 25.3 months (95% CI NA–NA) for stage IIIB. The median RFS was 34.3 months (95% CI 20.9–47.7 months) for stage I, 12.3 months (95% CI 9.5–15.1 months) for stage II, 13.7 months (95% CI 0–28.9 months) for stage IIIA and 6.9 months (95% CI NA–NA) for stage IIIB. These findings showed that early-stage cases, particularly cStage I, had favourable outcomes compared to cStage II and beyond (OS: *P* = 0.0016, RFS: *P* = 0.013). These results indicate a stronger correlation with prognosis when using the 9th edition of the TNM classification compared to the 8th edition (Fig. 3A and B).

The univariate analysis, which included age, sex, tumour location, procedure, histological subtype, clinical stage (8th and 9th editions) and postoperative adjuvant chemotherapy, revealed that the 8th edition clinical stage was not a significant prognostic factor for OS (*P* = 0.075) or RFS (*P* = 0.062). However, according to the 9th edition, clinical stage emerged as a significant prognostic factor for OS (*P* = 0.003) and RFS (*P* = 0.015). In the multivariable analysis incorporating the 9th edition clinical stage, clinical stage [hazard ratio (HR) 0.268, 95% CI 0.104–0.687; *P* = 0.006] and postoperative adjuvant therapy (HR 0.359, 95% CI 0.160–0.803; *P* = 0.013) were identified as independent prognostic factors for OS. Regarding RFS, clinical stage (HR 0.464, 95% CI 0.230–0.937; *P* = 0.032), postoperative adjuvant therapy (HR 0.450, 95% CI 0.212–0.953; *P* = 0.037) and histological type (HR 0.356, 95% CI 0.178–0.710; *P* = 0.003) were independent prognostic factors (Table 3). The AUC values for OS and RFS based on the 9th edition of the TNM classification were 0.763 (95% CI 0.644–0.882) and 0.622 (95% CI 0.477–0.768), respectively.

**Table 3:** Univariate and multivariable analyses of overall survival and relapse-free survival in patients with pleural mesothelioma

	Univariate			Multivariable		
Variables	Hazard ratio	95% CI	*P*-value	Hazard ratio	95% CI	*P*-value
Overall survival						
Model 1						
Age, <70 years (vs. ≥70 years)	0.479	0.241–0.954	0.036	0.489	0.242–0.988	0.046
Sex, female (vs. male)	0.886	0.269–2.919	0.842			
Tumour location, left (vs. right)	1.012	0.689–1.485	0.953			
Procedure, P/D (vs. extended P/D)	0.962	0.337–2.747	0.942			
Histological subtype, epithelioid (vs. sarcomatoid/biphasic)	0.322	0.157–0.659	0.002	0.428	0.196–0.936	0.034
Clinical stage (8th edition), IA (vs. IB-)	0.522	0.255–1.067	0.075			
Adjuvant chemotherapy, yes (vs. no)	0.287	0.140–0.588	<0.001	0.402	0.184–0.879	0.022
Model 2						
Age, <70 years (vs. ≥70 years)	0.479	0.241–0.954	0.036	0.586	0.280–1.226	0.156
Sex, female (vs. male)	0.886	0.269–2.919	0.842			
Tumour location, left (vs. right)	1.012	0.689–1.485	0.953			
Procedure, P/D (vs. extended P/D)	0.962	0.337–2.747	0.942			
Histological subtype, epithelioid (vs. sarcomatoid/biphasic)	0.322	0.157–0.659	0.002	0.518	0.238–1.129	0.098
Clinical stage (9th edition), I (vs. II-)	0.260	0.106–0.636	0.003	0.268	0.104–0.687	0.006
Adjuvant chemotherapy, yes (vs. no)	0.287	0.140–0.588	<0.001	0.359	0.160–0.803	0.013
Relapse-free survival						
Model 1						
Age, <70 years (vs. ≥70 years)	1.024	0.557–1.882	0.940			
Sex, female (vs. male)	0.861	0.306–2.419	0.776			
Tumour location, left (vs. right)	1.092	0.774–1.541	0.615			
Procedure, P/D (vs. extended P/D)	1.002	0.391–2.567	0.996			
Histological subtype, epithelioid (vs. sarcomatoid/biphasic)	0.277	0.140–0.547	<0.001	0.307	0.153–0.614	<0.001
Clinical stage (8th edition), IA (vs. IB-)	0.557	0.301–1.030	0.062			
Adjuvant chemotherapy, yes (vs. no)	0.440	0.214–0.903	0.025	0.547	0.263–1.139	0.107
Model 2						
Age, <70 years (vs. ≥70 years)	1.024	0.557–1.882	0.940			
Sex, female (vs. male)	0.861	0.306–2.419	0.776			
Tumour location, left (vs. right)	1.092	0.774–1.541	0.615			
Procedure, P/D (vs. extended P/D)	1.002	0.391–2.567	0.996			
Histological subtype, epithelioid (vs. sarcomatoid/biphasic)	0.277	0.140–0.547	<0.001	0.356	0.178–0.710	0.003
Clinical stage (9th edition), I (vs. II-)	0.439	0.225–0.854	0.015	0.464	0.230–0.937	0.032
Adjuvant chemotherapy, yes (vs. no)	0.440	0.214–0.903	0.025	0.450	0.212–0.953	0.037

CI: confidence interval; P/D: pleurectomy/decortication.

## DISCUSSION

Recent findings from the MARS2 trial have raised concerns about the effectiveness of curative intent surgery for PM. These results underscore the need for careful re-evaluation of treatment strategies and further assessment of patient selection criteria, as well as the potential benefits and risks of surgery. This study examined the surgical outcomes of P/D for patients with PM at our institution, revealing that good prognosis was associated with epithelioid histology and early-stage disease, emphasizing the role of surgical intervention in these patient subsets. Furthermore, we observed improved accuracy in identifying groups with favourable prognosis using the newly revised 9th edition of the TNM classification, making this classification a valuable tool for selecting candidates for surgery.

Our findings demonstrate a high safety profile of surgery, with only one 90-day perioperative death, despite a relatively high complication rate. Additionally, preserving the lung parenchyma enabled the administration of postoperative adjuvant chemotherapy at a high rate, consistent with previous reports from our institution [9]. Moreover, a high proportion of patients in our study achieved MCR, likely contributing to the favourable outcomes reported in the MARS2 trial [2] and real-world clinical experience in Japan [3]. Consistent with the findings of Nakamura *et al.* [10], these results underscore the significance of perioperative management and surgical proficiency in ensuring the success of P/D. Subtype analysis affirmed the known association between non-epithelioid subtypes and poor prognosis, highlighting the primary benefit of surgical intervention in patients with epithelioid histology. Further analysis by pathological stage demonstrated improved prognosis among early-stage cases, particularly pStage IA, emphasizing the clear benefit of surgery for these patients.

However, the correlation between clinical staging and favourable outcomes was less pronounced compared to pathological staging, possibly due to challenges in the accurate assessment of the T descriptor preoperatively using imaging [11]. Despite this, the revised 9th edition of the TNM classification offers improved T-descriptor assessment, leading to more precise selection of patients with good postoperative prognosis [4]. Our study’s univariate analysis revealed that the 8th edition of staging was not a significant prognostic factor for OS or RFS, whereas staging in the 9th edition was an independent prognostic factor for OS and RFS in multivariable analysis; this underscores the enhanced sensitivity of surgical candidacy evaluation. Therefore, surgical intervention should be considered for patients with the epithelioid subtype and thin pleura (cStage I).

**Figure 4:**
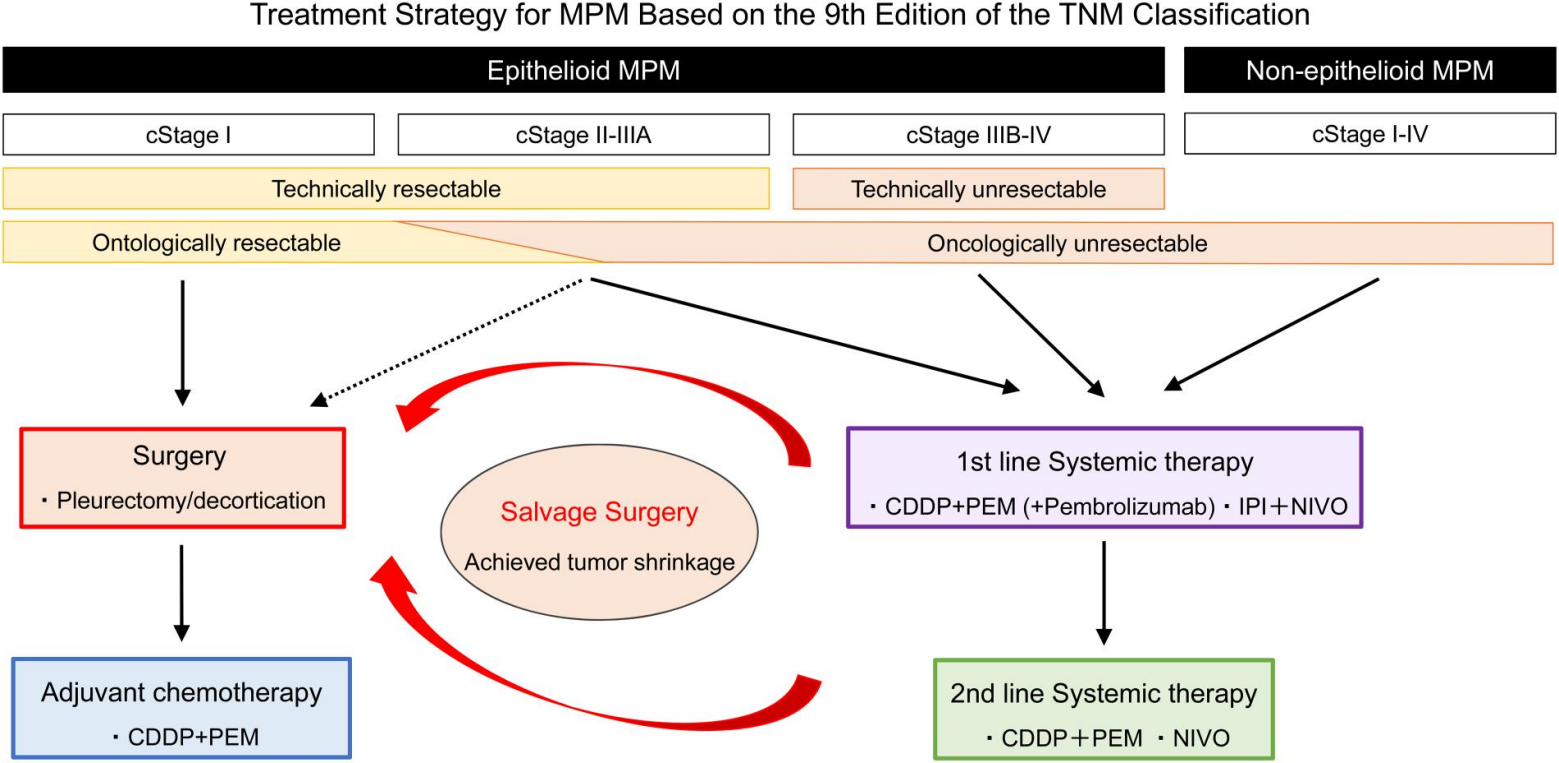
Treatment strategy based on the 9th edition of the TNM classification at our institution. Technically resectable: cases where macroscopic complete resection (MCR) can be achieved. Oncologically resectable: cases where MCR can be achieved and oncological control of the disease is possible.

Despite the improved outcomes for cStage II or IIIA cases compared to previous reports, a substantial proportion of patients experienced recurrence within a relatively short period postoperatively, suggesting that while these tumours are technically resectable, they are challenging to eradicate. Therefore, a strategy involving initial systemic therapy followed by surgery after achieving tumour shrinkage may be a suitable option for these patients. For stage IIIB or IV cases, or those with non-epithelioid histology, systemic therapy, including immunotherapy, is usually recommended. Nonetheless, our experience with salvage surgery has demonstrated promising outcomes, revealing a correlation between radiological tumour shrinkage and pathological response, indicating that surgical intervention may prolong survival in cases showing a positive response [12]. Thus, our institution proposes a treatment strategy based on the 9th edition of the TNM classification (Fig. 4).

### Limitations

This study has the following limitations. First, this was a retrospective cohort study, and the data were obtained from a single institution, which does not adequately reflect the characteristics of the general population of this disease. Second, the study period extended from 2012 to 2022, during which diagnostic and therapeutic algorithms for this disease evolved substantially, resulting in variable treatment strategies across the cohort. Third, we did not compare our findings with non-surgical cases, which limits the ability to assess the full spectrum of therapeutic options. Nonetheless, there was a patient with cStage IA (8th edition) and cStage I (9th edition) who survived >7 years without relapse after surgery and without adjuvant therapy, owing to the introduction of home oxygen therapy, thus supporting the potential benefit of surgical intervention.

Despite these limitations, our findings demonstrate the safety and therapeutic efficacy of P/D, particularly in early-stage and epithelioid PM cases. The endorsement of cStage I in the new 9th edition of the TNM classification as a criterion for selecting surgical candidates is supported by our observations.

## CONCLUSION

In conclusion, while our retrospective study provides valuable insights into the potential role of surgery for patients with resectable PM, it is essential to recognize that further research is necessary to establish definitive survival benefits. We emphasize the importance of careful patient selection based on histological subtype and accurate staging using the revised TNM classification. These factors may optimize treatment strategies and potentially improve outcomes for this challenging disease. The newly revised 9th edition of the TNM classification aims to enhance the precision of surgical candidate selection, which could lead to better patient prognoses.

## Data Availability

The datasets generated and analysed during the current study are available from the corresponding author.

## ABBREVIATIONS

AUC: Area under the curve
CI: Confidence interval
HR: Hazard ratio
MCR: Macroscopic complete resection
OS: Overall survival
P/D: Pleurectomy/decortication
PM: Pleural mesothelioma
RFS: Relapse-free survival
TNM: tumour–node–metastasis

## References

[1] Popat S , Baas P, Faivre-Finn C et al; ESMO Guidelines Committee. Electronic address: clinicalguidelines@esmo.org. Malignant pleural mesothelioma: ESMO clinical practice guidelines for diagnosis, treatment and follow-up. Ann Oncol 2022;33:129–42. 34861373 10.1016/j.annonc.2021.11.005

[2] Lim E , Waller D, Lau K et al; MARS 2 Investigators. Extended pleurectomy decortication and chemotherapy versus chemotherapy alone for pleural mesothelioma (MARS 2): a phase 3 randomised controlled trial. Lancet Respir Med 2024;12:457–66. 38740044 10.1016/S2213-2600(24)00119-X PMC11136673

[3] Hasegawa S , Shintani Y, Takuwa T et al Nationwide prospective registry database of patients with newly diagnosed untreated pleural mesothelioma in Japan. Cancer Sci 2024;115:507–28. 38047872 10.1111/cas.16021 PMC10859622

[4] Gill RR , Nowak AK, Giroux DJ et al; members of the International Association for the Study of Lung Cancer Staging and Prognostic Factors Committee, Advisory Boards and Participating Institution. The IASLC mesothelioma staging project: proposals for revisions of the “T” descriptors in the forthcoming ninth edition of the TNM classification for pleural mesothelioma. J Thorac Oncol 2024;19:1310–25. 38521202 10.1016/j.jtho.2024.03.007 PMC11380601

[5] Rusch VW , Chansky K, Kindler HL et al; IASLC Staging and Prognostic Factors Committee, advisory boards, and participating institutions. The IASLC mesothelioma staging project: proposals for the M descriptors and for revision of the TNM stage groupings in the forthcoming (eighth) edition of the TNM classification for mesothelioma. J Thorac Oncol 2016;11:2112–9. 27687962 10.1016/j.jtho.2016.09.124

[6] Tanaka F , Imanishi N, Takenaka M, Taira A. Non-incisional pleurectomy-decortication for malignant pleural mesothelioma. Surg Today 2018;48:656–8. 29492684 10.1007/s00595-018-1624-0

[7] Tanaka F , Takenaka M, Imanishi N et al Non-incisional pleurectomy/decortication for malignant pleural mesothelioma. Gen Thorac Cardiovasc Surg 2021;69:1320–5. 34028663 10.1007/s11748-021-01643-z

[8] Dindo D , Demartines N, Clavien PA. Classification of surgical complications: a new proposal with evaluation in a cohort of 6336 patients and results of a survey. Ann Surg 2004;240:205–13. 15273542 10.1097/01.sla.0000133083.54934.ae PMC1360123

[9] Kanayama M , Mori M, Matsumiya H et al Surgical strategy for malignant pleural mesothelioma: the superiority of pleurectomy/decortication. Surg Today 2022;52:1031–8. 35044520 10.1007/s00595-021-02437-9

[10] Nakamura A , Hashimoto M, Kuroda A et al Impact of operation on disease progression and survival of patients with pleural mesothelioma. Ann Thorac Surg 2024;118:216–23. 38428631 10.1016/j.athoracsur.2024.02.022

[11] Gill RR , Yeap BY, Bueno R, Richards WG. Quantitative clinical staging for patients with malignant pleural mesothelioma. J Natl Cancer Inst 2018;110:258–64. 29931180 10.1093/jnci/djx175 PMC6009654

[12] Takenaka M , Kuroda K, Yoshimatsu K et al Salvage pleurectomy/decortication following immunotherapy for malignant pleural mesothelioma. Interdiscip Cardiovasc Thorac Surg 2024;38:ivad173. 37966912 10.1093/icvts/ivad173 PMC10859178

